# The Variation of *Oncidium* Rosy Sunset Flower Volatiles with Daily Rhythm, Flowering Period, and Flower Parts

**DOI:** 10.3390/molecules22091468

**Published:** 2017-09-04

**Authors:** Yi-Tien Chiu, Hsin-Chun Chen, Chen Chang

**Affiliations:** 1 Department of Horticulture, National Chung Hsing University, 145 Xingda Rd., South Dist., Taichung 402, Taiwan; o_oten@yahoo.com.tw; 2 Department of Cosmeceutics, China Medical University, 91 Hsueh-Shih Rd., Taichung 404, Taiwan

**Keywords:** fragrance, *Oncidium*, headspace solid-phase microextraction (HS-SPME), flowering period, daily rhythm, perianth

## Abstract

*Oncidium* is an important ornamental crop worldwide, and in recent years, the characteristics of the flower aroma have become a concern for breeders. This study used headspace solid-phase microextraction (HS-SPME) and gas chromatography/mass spectrometry (GC-MS) analysis of the volatile compounds to study the aroma characteristics of *Onc.* Rosy Sunset. A total of 45 compounds were identified, with the major compound being linalool. *Onc.* Rosy Sunset had the highest odor concentration from 10:00 to 12:00 and lowest from 20:00 to 24:00. The inflorescence emitted the highest quantities of volatile compounds during stages 3–6, which then decreased with the aging of the flowers. In *Onc.* Rosy Sunset, the sepals and petals were the major parts for the floral fragrance emission, in which linalool content was the highest, whereas the lip and column had a different composition of major volatile compounds, of which benzaldehyde, β-myrcene, and β-caryophyllene dominated.

## 1. Introduction

Oncidiinae are native to the Neotropical region, include approximately 87 genera, with 1600 species, varieties or cultivars, and have become economically important ornamental crops worldwide for use in the cut-flower and pot-plant industries [1,2,3]. Floral fragrance is one of the important features of the ornamental orchid, and can improve the aesthetic value, quality of flower products, and their economic merit. Cultivating fragrance is a current trend in orchid breeding, with several studies involving orchid volatile compound analysis and synthesis, mainly focused on *Phalaenopsis* [4,5,6,7,8]. There are few fragrant cultivars of *Oncidium* at present, with the minimal research into their aromatic volatile compounds mostly confined to local journals and focused on *Onc.* Sharry Baby, a large potted plant [9,10,11,12].

Floral fragrance is also a key factor in plant interactions with insects and is important for plant reproduction and evolution [13,14]. The pollinator plays a key role with orchid sexual propagation; therefore, fragrance is especially important for orchids [5].

The extraction of volatile compounds from flowers is an important step in the analysis of floral aroma. The typical methods used include liquid–liquid extraction, purge and trap, steam distillation (SD), simultaneous distillation extraction (SDE), supercritical-fluid extraction (SFE), solid-phase extraction (SPE), and solid-phase microextraction (SPME) [15]. SPME is simple, fast, sensitive, requires no solvent [16], and has been successfully used to analyze volatile compounds in a variety of flowering plants, such as *Hosta*, Tree peony, *Styrax tonkinensis*, *Narcissus tazetta*, and *Phalaenopsis* [8,15,17,18,19]. Many of these floral fragrance volatiles are terpenes, phenylpropanoids, or fatty acid derivatives [5,13,14].

It is useful to investigate the composition and variation of the aroma of these plants for future research into the mechanism of aroma volatiles or for breeding fragrant cultivars of *Oncidium*. *Onc.* Rosy Sunset is a new breed of medium-sized potted orchid with a strong aroma, multiple stalks, and a perfect size for a potted plant, which is a good candidate for the breeding of ornamental or fragrant flowers in the future. This study analyzed the volatile components throughout the flowering period and from different flower parts. It is also the first study of how the volatiles vary during the daily rhythm for *Oncidium*.

## 2. Results and Discussion

### 2.1. Analysis of the Volatiles in Onc. Rosy Sunset Flowers

**Figure 1:**
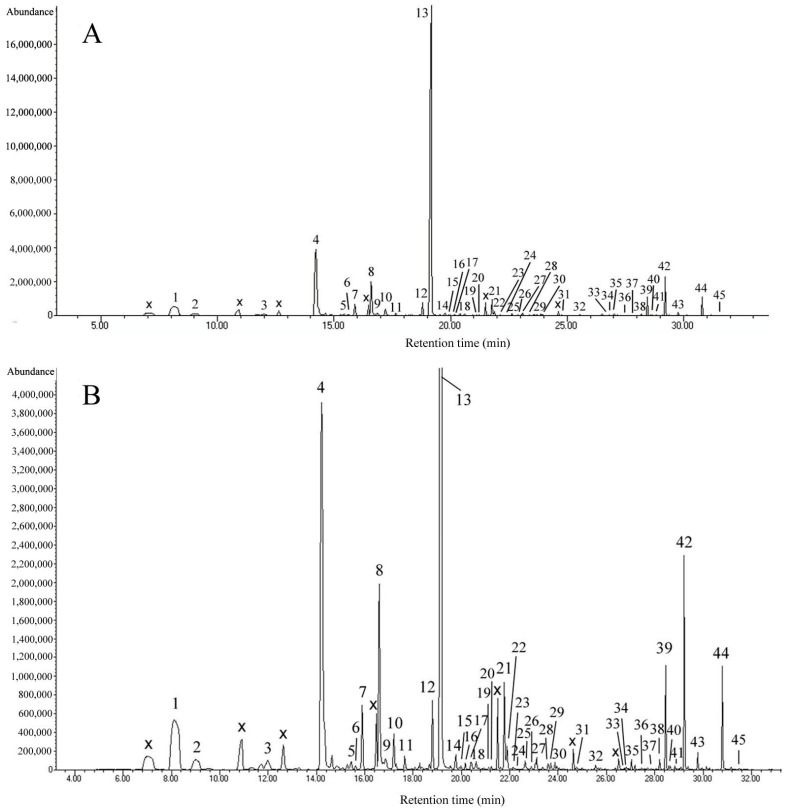
Total ion chromatogram of volatile components of *Onc.* Rosy Sunset (peak numbering in accordance with numbering in Table 1; x, column, or septum bleed). (**A**) Original total ion chromatogram; (**B**) Normalized total ion chromatogram.

According to the total ionic chromatogram (Figure 1), 45 volatile compounds were identified, including 22 terpenes, 7 aromatics or benzene derivatives, 3 aliphatics, 6 other hydrocarbons, and 7 other compounds (Table 1). *Onc.* Rosy Sunset had a complex aroma volatile composition; however, more than half of these volatile compounds are terpenes of the type monoterpene and sesquiterpene. Most plant volatiles fall into the terpenoid or phenylpropanoid/benzenoid classes of compounds, or are fatty acid derivatives, which are used by terpenes to synthesize the mevalonic acid (MVA) and methylerythritol phosphate (MEP) pathways [13,14]. According to the isolation method, the environmental conditions of the experiment and variations of the cultivars will create a different volatile composition of *Oncidium*. A comparison between the volatiles compounds of *Onc.* Rosy Sunset with other *Oncidium* cultivars is shown in Table 1 [9,10,11,12].

The main constituent of the volatile compounds was linalool (52.8%), an important aroma component of *Onc.* Rosy Sunset. This was followed by benzaldehyde (9.25%), β-myrcene (4.15%), and tiglaldehyde (3.65%); benzyl alcohol and nerolidol were 2.14% and 2.11%, respectively, and other constituents of relatively low content were below 2% (Table 1). Linalool emits a sweet floral, fragrance, which also exists in a number of essential oils such as ho-leaf, bois de rose, lavender, and coriander oils [20,21]. Hsiao et al. [6] investigated the role of geranyl diphosphate synthase (*GDPS*) in the orchid aroma composition and emission. The highest levels of *GDPS* gene expression were concomitant with maximal emission of geraniol and linalool in *Phalaenopsis*. In the aroma components of several *Oncidium* flowers, alcohols of terpene derivatives are found to dominate; for instance, in *Onc.* Sharry Baby, geraniol and linalool were among the main constituents [9]. This demonstrates that the *GDPS* gene may also play an important role in *Oncidium* aroma components.

### 2.2. Changes of Volatile Components with Daily Rhythm

Table 2 shows that the volatile components changed with the daily rhythm. The total relative content is expressed in total peak areas. The odor was strongest at 12:00, with a total peak area of 5117, followed by 4555 at 10:00, and it gradually declined until 18:00–04:00, with the lowest values of 535–640, and then rose again at 8:00 with a peak area of 1917. These results were similar to those reported for *Phal.* Nobby’s Pacific Sunset and Gongora bufonia, which showed stronger odor levels at 09:00–13:00 and 10:00, respectively [8,22]. Orchids possess entomophilous flowers, and the aroma volatile components are key for the attraction of insects. Flowers emit different volatile compositions at different times to attract specific pollinators, such as a specific type of bee or diurnal/nocturnal insects [23,24]. The diurnal bee is a pollinator for both Phalaenopsis and Gongora bufonia; therefore, the strongest odor emitted was during the day.

There were significant differences in the emission of most single compounds. The exceptions were tiglaldehyde, 2-methyl-2-buten-1-ol, benzaldehyde, β-myrcene, 2-ethylhexanol, methyl salicylate, 2-ethylhexyl acrylate, β-citral, β-caryophyllene, (*Z*)-β-farnesene, benzyl tiglate, and nerolidol, for which no obvious differences in relative percentage of floral scent emitted over a daily rhythm were found. Linalool had the highest relative percentage change, with a maximum of 37.09–35.8% at 10:00–12:00 and a minimum of 4.72% at 22:00 (Table 2). Such changes were similar to the total relative content changes over the daily rhythm. We note that Borg-Karlson et al. [25] studied linalool as a mate attractant component in the male bee. Not all the volatile compositions peaked during the day, as the relative percentages of β-pinene, limonene, (*E*)-4,8-dimethyl-l,3,7-nonatriene, menthol, tridecane, and copaene were highest at 18:00–22:00. The emission of *cis*-linalool oxide, α-terpineol, isopentyl benzoate, and 2-methylpropanoate peaked at nightfall (16:00–18:00; Table 2). Dötterl et al. [23] suggested that changes in aroma components during the daily rhythm of *Silene otites* may attract specific insects at different times, not only the originally identified moths and mosquitoes. This may explain why some volatile components were highest at nightfall or nighttime for *Onc.* Rosy Sunset. It is worth noting that (*E*)-4,8-dimethyl-l,3,7-nonatriene, which is generally emitted from the damaged leaves and attracts insects, is only found in a few flowers, such as *Magnolia* taxa and *Liriodendron tulipifera* [26]. In our results, (*E*)-4,8-dimethyl-l,3,7-nonatriene was emitted in highest concentrations at nighttime, although it is not known whether this is related to the attraction of nocturnal insects. Linalool was a primary component of emission, over all times of the daily rhythm.

In terms of horticultural characteristics, the finding that the *Onc.* Rosy Sunset odor was strongest in the morning can be used to market the flowers to consumers and as a reference for hybrid offspring selection time. In addition, the selection of parent plants that emit aroma during the afternoon or nightfall can be used in breeding programs with *Onc.* Rosy Sunset to breed new *Oncidium* cultivars that emit aroma all day.

### 2.3. Changes of Volatile Components with the Flowering Period

**Figure 2:**
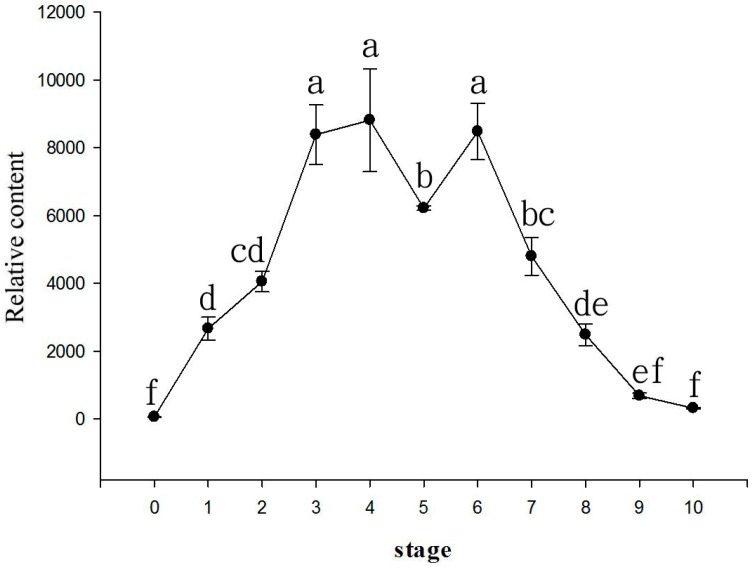
Changes in the volatile components of *Onc.* Rosy Sunset during the flowering period (Different letters indicate significant differences at *p* < 0.05 by least significant difference LSD multiple range).

The quantity of volatile components increased with the flowering period, peaking during stages 3–6 of the inflorescence and decreasing after stage 7, with almost no odor at stage 9 (Figure 2). The level of emitted volatile components changes with the flowering period, with the strongest odor during stages 3–6 in *Onc.* Rosy Sunset and not in the initial flowering stage. This result was repeated in a variety of flowers. For comparison, the flowering period of *Phal.* Nobby’s Pacific Sunset was 51 days, and the odor was strongest at 8–20 days after flowering (DAF) [8]. *Phal. bellina* emitted the highest levels of geraniol and linalool at 5–7 DAF, and the flowering period was 14 days [6]. The odor was strongest at 2 or 3 DAF for *Narcissus tazetta* var. *chinensis* with a 6-day flowering period [17].

**Figure 3:**
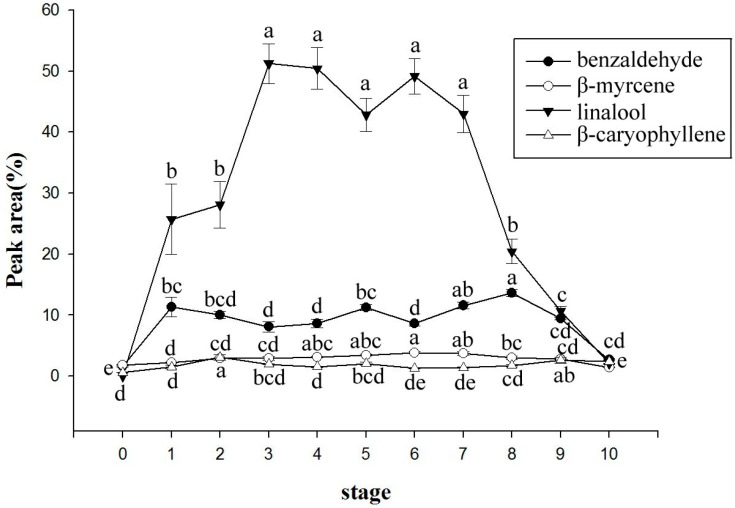
Changes in the major volatile components of *Onc.* Rosy Sunset during the flowering period (Different letters indicate significant differences at *p* < 0.05 by LSD multiple range).

Four of the major volatile compounds were linalool, benzaldehyde, β-myrcene, and β-caryophyllene, and their relative percentage change in composition with the flowering period is shown in Figure 3. The relative percentage of linalool was much higher than that of other volatile components during all phases of the *Onc.* Rosy Sunset flowering period, and the level significantly increased during the first flower bloom of the inflorescence. These changes were similar to those in the total relative content. The relative percentage of benzaldehyde was higher during stages 7–8, which may be affected by the decrease in the relative quantity of linalool. No significant changes of β-myrcene and β-caryophyllene were observed during the flowering period (Figure 3). *Onc.* Sharry Baby had a higher quantity of volatile compounds during the flowering stage than in the initial flowering stage, and the relative content of alkenes, alcohol, and esters was found to increase with the flowering stage [10]. However, the total relative content significantly lowers at stage 5 in our experiment, but rises again at stage 6, presumably due to the colder weather. Although the experiment temperature was controlled, the environment still varied in temperature because of a cold snap. Zhang et al. [12] reported that the temperature will affect the volatile emissions in *Onc.* Sharry Baby, and the amount of aroma components and total relative content were both higher at 30 °C than at 10 °C. Adachi et al. [22] also indicated that for *Gongora bufonia*, the odor was strongest at 10:00, which was the period in which the temperature began to rise.

### 2.4. Volatile Components in Different Parts of Onc. Rosy Sunset Flowers

The *Onc.* Rosy Sunset flowers were divided into petal, sepal, lip, and column parts to analyze the differences in their volatile emission profile compositions. The results showed that the total relative content and amounts of volatile compounds in the petals and sepals were significantly higher than in the lip and column. A total of 43 compounds were identified from the petals and sepals, with a total relative content count of 12,798 and 11,120, respectively. A total of 27 and 25 compounds were found from the lip and column regions, respectively, with the total relative content count at 859 and 716, respectively (Table 3). *Phal. bellina* emitted odor from the perianth and not the column [5]. In flowering plants, scent glands are called osmophores. In the orchid, odors are not produced by all flower parts, however, and the osmophores are found on the sepals, petals, or parts of the lip, mainly at the base of the lip [27,28], as seen in *Vanilla edwallii* and *Gongora bufonia* [22,29]. The most significant structure of *Oncidium* flowers is the lip, and the callus at the base of the lip is often considered the location of the osmophores. Zhang et al. [10] reported that the lip of *Onc.* Sharry Baby was the location where the majority of the odor was emitted.

**Figure 4:**
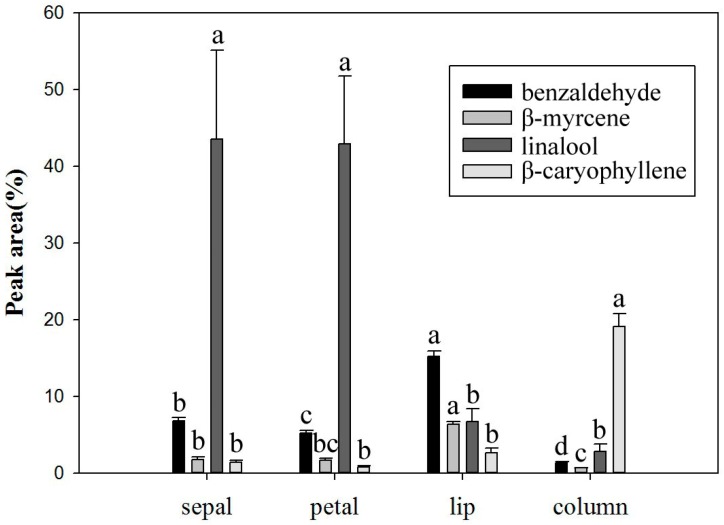
Changes in the major volatile components of different parts of *Onc.* Rosy Sunset (Different letters indicate significant differences at *p* < 0.05 by LSD multiple range).

Although hydrocarbons were the most common type of volatile compounds from the four flower parts, alcohols had the highest total relative content in the petals and sepals (Table 3). As shown in Figure 4, the major component found in the petals and sepals was linalool (classed as an alcohol), which was shown to be the strongest odor in two parts. However, the strongest odor was detected from the lip of *Onc.* Sharry Baby, and the column had the most complex volatile compounds [10]. The major volatile compounds were also found to differ between the four parts, with linalool found as the major compound in the petals and sepals; benzaldehyde and β-myrcene had the highest relative percentage in the lip, while β-caryophyllene was the highest in the column (Figure 4). Meanwhile, *Phal.* Nobby’s Pacific Sunset was reported to have the highest total relative content of volatile compounds in the petals and dorsal sepals. The major volatile compound in all five flower parts studied was linalool [8]. There is, however, a different distribution of major volatile compounds in different flower parts of *Onc.* Rosy Sunset. It is thought that the location of the osmophores and structure changes between the orchid species and different cultivars, even in *Oncidium*, creates a large effect.

In this study, the results showed that daily rhythm, flowering period, and temperature affected aroma emissions. These results were also showed in *Onc.* Sharry Baby; the odor was strongest during the flowering stage and in a 30 °C environment, on the other hand, the lower temperature reduced the number of volatile compounds and relative content [10,12]. The qualitative and quantitative differences between the present study and those from other parts of the world may be attributable to the differences in various extraction techniques, cultivated varieties, and ecological and climatic conditions [30]. Therefore, we set the conditions at 25 °C, 5–8 DAF, and extraction was performed during the 10:00–12:00 AM period for the more stable analytical performance.

## 3. Materials and Methods

### 3.1. Plant Materials

*Onc.* Rosy Sunset is an ornamental potted *Oncidium* with a sweet scent, which was purchased from Yung Hsin Orchids, Taichung, Taiwan. Volatile components were identified during the flowering period, cultivated in the National Museum of Natural Science cold room greenhouse, controlled at 25 ± 3 °C.

### 3.2. Volatile Components of Onc. Rosy Sunset

The volatile compounds extracted from the *Onc.* Rosy Sunset flowers were analyzed by headspace solid-phase microextraction (HS-SPME) coupled with gas chromatography (GC) and gas chromatography-mass spectrometry (GC-MS). In a study using five different SPME fibers, volatile compounds were analyzed in *Phal.* Nobby’s Pacific Sunset. The results show that fibers with 50/30 μm divinylbenzene/carboxen/polydimethylsiloxane (DVB/CAR/PDMS) had optimal extraction ability, with linalool as one of the major volatile compounds [8]. This study also used 50/30 μm DVB/CAR/PDMS fibers for better analytical performance.

**Figure 5:**
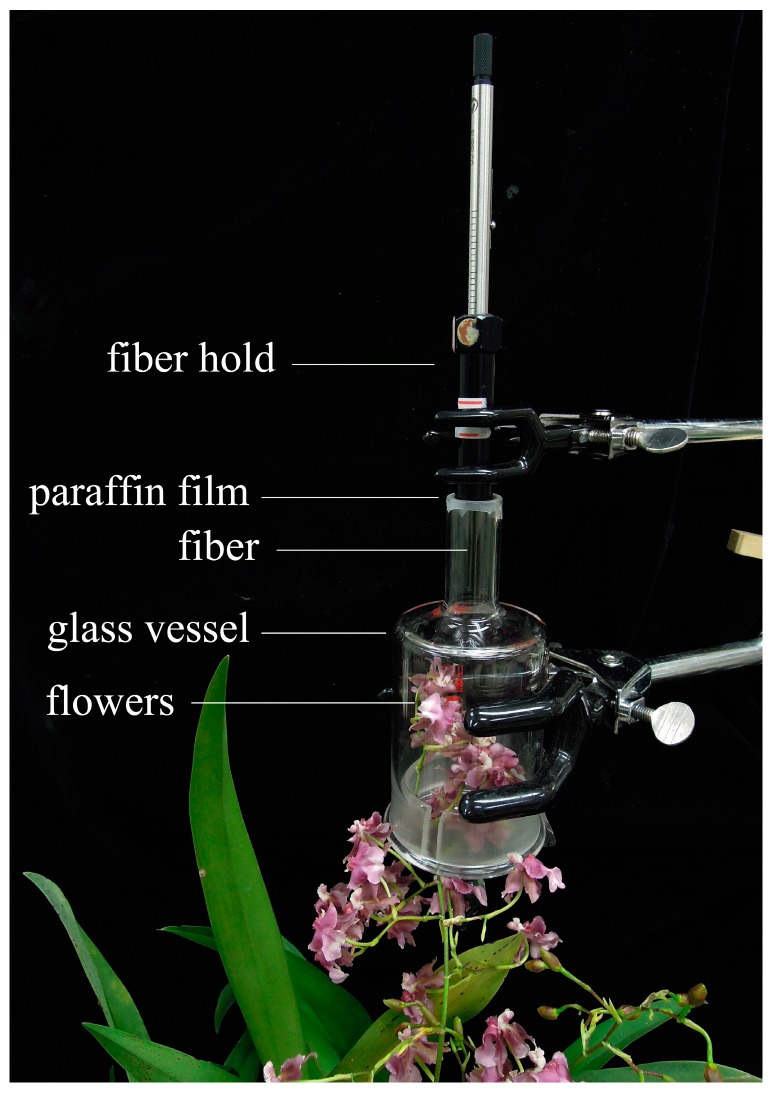
Diagram of enclosure system for collection of floral aroma from *Onc.* Rosy Sunset.

HS-SPME of volatile components in *Onc.* Rosy Sunset was performed using fiber adsorption techniques, and subsequent analysis was carried out using GC (gas chromatography, 6890 GC, Agilent, Santa Clara, CA, USA) and GC-MS (gas chromatography-mass spectrometry, 6890 GC-5973N MSD, Agilent, Santa Clara, CA, USA). At 10:00–12:00 AM *Onc.* Rosy Sunset inflorescence with 10 flowers, 5-8 DAF, was enclosed with a glass vessel (9 cm × 6.5 cm) and sealed with paraffin film, the device is shown in Figure 5. SPME fiber (50/30 μm DVB/CAR/PDMS, Supelco, Bellefonte, PA, USA) was exposed to the inflorescence for 40 min in order to extract the aroma components in a 25 ± 3 °C air conditioned laboratory, after which each sample was injected into a gas chromatograph injection unit. This experiment and all other experiments in this study were carried out in triplicate.

### 3.3. Changes of Volatile Components with Daily Rhythm

The *Onc.* Rosy Sunset inflorescence with 10 flowers, 5–8 DAF, was collected and analyzed for volatile components at two hour intervals between 10:00 and 08:00 (inclusive). The SPME method, as described above, was carried out for each period in triplicate, and each sample monitored the changes for 24 h. During non-adsorption times, the paraffin film was opened to ventilate the glass vessel.

### 3.4. Volatile Component Changes during the Flowering Period

For the flower buds of the *Onc.* Rosy Sunset, 10 flower inflorescences were selected and defined as follows: stage 0, flower buds turned color; stage 1, 50% of flowers in bloom; stage 2, full inflorescence flowering; stage 3–8, every 2 days that have elapsed post flowering; stage 9, the first withered flower observed; and stage 10, 50% of flowers withered. The SPME method, as described above, was performed during the 10:00–12:00 AM period.

### 3.5. Volatile Components in Different Parts of the Onc. Rosy Sunset Flowers

The 5–8 DAF flowers were picked and separated into four parts: petal, sepal, lip, and column. The four parts of ten flowers were separated into glass bottles (precleaned # 27343 22-mL clear screw cap vials; Supelco Inc.). The SPME method was used to adsorb volatiles for 60 min.

### 3.6. GC and GC-MS

A GC equipped with a 60 m × 0.25 mm i.d column was used to perform qualitative and quantitative analysis of the volatile compounds. The column used a DB-1 fused-silica capillary with a film thickness of 0.25 μm and a flame ionization detector. The injector and detector temperatures were maintained at 250 and 300 °C, respectively. The sample was held at 40 °C for 1 min and raised to 200 °C at 2 °C/min then held for 9 min at the temperature of oven. The nitrogen carrier gas flow rate was 1 mL/min. Kovats indices were calculated for the separated components relative to a C5–C25 *n*-alkanes mixture [31]. The peak area normalization measurements were used to calculate the percentage composition.

The volatile compounds were identified using a GC-MS. The GC was equipped as described above and a DB-1 fused-silica capillary column with a film thickness of 0.25 μm coupled to an MS. The injector temperature was held at 250 °C. The helium carrier gas flow rate was 1 mL/min. The ionization energy was 70 eV at 230 °C. A mass spectrometry (MS) library (Wiley 7N) was used to identify the constituents. Comparison of the GC and GC-MS analysis results was completed to identify compounds qualitatively and quantitatively. In addition, the constituents were confirmed by comparing the Kovats indices or GC retention time data with those of authentic standards or published in the literature.

### 3.7. Statistical Analysis

The ANOVA mean value among treatments was compared with the LSD multiple range at a 5% level of significance. This was performed using CoStat6.1 software (CoHort software, Minneapolis, Mn, USA).

## 4. Conclusions

The major aroma volatile compounds of *Onc.* Rosy Sunset were linalool, benzaldehyde, β-myrcene, tiglaldehyde, benzyl alcohol, and nerolidol. The odor was strongest around noon three days after full flowering, and the main source of aroma was from the petals and sepals. This paper is the first comprehensive study of the change in volatile aroma compounds with daily rhythm, flowering period, and different flower parts for *Oncidium*. In the application of horticulture, this is useful information for commodity sales, understanding the breeding of parental characteristics such as aroma composition, and as a reference for future research on the location and structural characteristics of osmophores in *Oncidium*.

## Figures and Tables

**Table 1:** Percentages of volatile compounds in *Onc.* Rosy Sunset.

Peak ^z^	RI ^y^	RI ^x^	Compound	Content (%)	References
1	718	713	tiglaldehyde	3.65 ± 0.70 ^w^	
2	754	761	2-methyl-2-buten-1-ol	1.72 ± 0.31	
3	874	856	*o*-xylene	0.26 ± 0.06	
4	931	930	benzaldehyde	9.25 ± 0.45	[8,17]
5	968	963	sabinene	0.41 ± 0.02	[17,18,21,24]
6	975	971	β-pinene	0.37 ± 0.02	[9,12,17,18,21,23,24]
7	983	979	β-myrcene	4.15 ± 0.13	[8,10,12,15,18,21,26]
8	1011	995	benzyl alcohol	2.14 ± 0.14	[17]
9	1017	1009	2-ethyl-1-hexanol	0.52 ± 0.07	
10	1022	1016	limonene	1.66 ± 0.02	[8,9,15,17,18,24,26]
11	1038	1031	(*E*)-β-ocimene	0.79 ± 0.03	[10,12,15,17,18,23,24,26]
12	1067	1064	*cis*-linalool oxide	1.47 ± 0.26	[23]
13	1085	1081	linalool	52.80 ± 2.75	[8,9,12,17,18,23,26]
14	1104	1097	(*E*)-4,8-dimethyl-l,3,7-nonatriene	0.14 ± 0.01	[8,26]
15	1116	1110	butyl tiglate	0.17 ± 0.02	
16	1117	1121	alloocimene	0.38 ± 0.02	[9,18]
17	1128	1124	camphor	0.20 ± 0.03	
18	1159	1144	2-ethylhexyl acetate	0.24 ± 0.04	
19	1150	1153	epoxylinalol	0.10 ± 0.01	
20	1163	1157	menthol	0.48 ± 0.06	
21	1170	1167	methyl salicylate	0.82 ± 0.18	
22	1172	1176	α-terpineol	1.04 ± 0.14	[17]
23	1185	1183	decanal	0.10 ± 0.01	
24	1200	1200	dodecane	<0.01	[12,18]
25	1201	1196	benzenepropanol	0.12 ± 0.01	
26	1215	1209	2-ethylhexyl acrylate	0.24 ± 0.01	
27	1220	1216	β-citral	0.70 ± 0.05	[10,15]
28	1232	1236	nerol	0.19 ± 0.02	[8,10,11,12,15,21]
29	1237	1244	geranial	0.40 ± 0.05	[8]
30	1249	1253	methyl hydrocinnamate	0.08 ± 0.01	
31	1275	1279	cinnamyl alcohol	<0.01	
32	1300	1300	tridecane	0.16 ± 0.02	[9,12,15]
33	1337	1348	butyl benzoate	<0.01	
34	1354	1362	vanillin	0.05 ± 0.01	[17]
35	1366	1367	benzyl 3-methylbutanoate	0.31 ± 0.02	
36	1373	1380	copaene	0.21 ± 0.01	[11,12,21]
37	1400	1400	tetradecane	0.06 ± 0.01	[12,18]
38	1429	1415	isopentyl benzoate	0.18 ± 0.02	
39	1432	1431	β-caryophyllene	1.41 ± 0.13	[12,15,17,21,26]
40	1444	1446	(*Z*)-β-farnesene	0.42 ± 0.03	[8,21]
41	1459	1455	2,6-di-tert-butylquinone	0.05 ± 0.01	
42	1474	1477	benzyl tiglate	1.17 ± 0.15	
43	1500	1498	β-bisabolene	0.20 ± 0.02	[17]
44	1548	1547	nerolidol	2.11 ± 0.25	[8,11,26]
45	1591	1582	2-methylpropanoate	0.17 ± 0.02	
			Total	91.12 ± 1.21	

^z^ peak number in accordance with the peak numbering in Figure 1; ^y^ RI: literature retention indices references were checked for all compounds on DB-1 column; ^x^ RI: retention indices obtained using series of *n*-alkanes (C5–C25) on DB-1 column; ^w^ data are mean ± SD of three replicates.

**Table 2:** Total volatile components and relative proportion of single compounds (%) in *Onc.* Rosy Sunset over a daily rhythm.

RI	Compound	10:00	12:00	14:00	16:00	18:00	20:00	22:00	24:00	02:00	04:00	06:00	08:00
Total amount		4555 ± 186 b ^Z^	5117 ± 387 a	2838 ± 106 c	1719 ± 174 de	991 ± 126 fg	640 ± 14 1g	535 ± 133 g	584 ± 114 g	741 ± 115 fg	934 ± 166 fg	1215 ± 151 ef	1917 ± 75 d
relative proportion %													
713	tiglaldehyde	9.18 ± 2.07	5.69 ± 0.99	9.01 ± 1.84	10.79 ± 1.57	9.31 ± 0.56	7.53 ± 0.70	6.84 ± 0.95	11.18 ± 3.58	12.83 ± 3.59	14.60 ± 3.46	14.86 ± 2.86	13.17 ± 2.66
761	2-methyl-2-buten-1-ol	2.91 ± 0.63	2.58 ± 0.27	4.09 ± 0.71	4.62 ± 0.30	4.92 ±0.80	7.51 ± 1.17	6.98 ± 1.38	10.35 ± 0.23	7.68 ± 2.12	9.25 ± 3.12	9.13 ± 2.83	7.58 ± 2.40
856	*o*-xylene	0.78 ± 0.09 c	1.57 ± 0.03 b	1.67 ± 0.06 a	0.74 ± 0.02 c	-	-	-	-	-	-	-	0.54 ± 0.01 d
930	benzaldehyde	6.64 ± 1.20	6.66 ± 1.19	8.38 ± 1.40	6.81 ± 0.58	6.44 ± 0.69	6.03 ± 0.42	5.74 ± 0.16	6.56 ± 0.77	5.66 ± 0.43	5.20 ± 0.41	5.94 ± 0.45	6.99 ± 0.79
963	sabinene	0.27 ± 0.01 c	0.24 ± 0.02 d	0.26 ± 0.01 cd	<0.01	-	-	-	-	-	<0.01	0.62 ± 0.01 a	0.43 ± 0.02 b
971	β-pinene	0.37 ± 0.04 fg	0.30 ± 0.02 g	0.39 ± 0.02 efg	0.51 ± 0.01 de	0.79 ± 0.07 ab	0.91 ± 0.04 a	<0.01	<0.01	<0.01	0.68 ± 0.05 bc	0.60 ± 0.04 cd	0.49 ± 0.04 def
979	β-myrcene	2.72 ± 0.19	2.60 ± 0.31	2.16 ± 0.13	2.03 ± 0.23	2.89 ± 0.56	3.66 ± 0.80	3.59 ± 0.66	4.37 ± 1.16	3.59 ± 0.93	3.31 ± 0.83	3.12 ± 0.46	3.14 ± 0.22
995	benzyl alcohol	2.11 ± 0.68 a	2.16 ± 0.56 a	2.40 ± 0.50 a	1.44 ± 0.19 ab	0.75 ± 0.11 bc	-	-	-	-	0.78 ± 0.01 bc	1.04 ± 0.23 b	1.61 ± 0.40 ab
1009	2-ethylhexanol	12.20 ± 11.47	11.08 ± 10.54	11.09 ± 10.61	12.39 ±11.76	12.73 ± 11.88	21.79 ± 16.71	<0.01	23.04 ± 17.65	14.37 ± 12.97	13.35 ± 12.08	11.15 ± 10.13	8.48 ± 7.68
1016	limonene	1.26 ± 0.08 cd	1.24 ± 0.08 cd	1.34 ± 0.23 bcd	1.14 ± 0.06 d	1.66 ± 0.20 abcd	1.96 ± 0.25 ab	1.92 ± 0.16 ab	2.20 ± 0.34 a	1.82 ± 0.25 abc	1.51 ± 0.11 bcd	1.72 ± 0.31 abcd	1.68 ± 0.25 abcd
1031	(*E*)-β-ocimene	0.51 ± 0.05 a	0.46 ± 0.06 a	0.31 ± 0.01 b	<0.01	-	-	-	-	-	-	-	0.43 ± 0.04 a
1064	*cis*-linalool oxide	0.85 ± 0.08 d	1.75 ± 0.19 c	2.59 ± 0.18 ab	2.70 ± 0.05 a	2.32 ± 0.07 b	1.40 ± 0.19 c	0.96 ± 0.19 d	0.89 ± 0.06 d	0.76 ± 0.04 d	0.67 ± 0.06 d	0.72 ± 0.10 d	0.95 ± 0.12 d
1081	linalool	35.8 ± 2.09 a	37.09 ± 3.15 a	21.50 ± 1.23 c	10.17 ± 0.64 e	7.74 ± 1.89 ef	5.73 ± 1.20 ef	4.72 ± 0.77 f	6.41 ± 1.47 ef	7.34 ± 1.48 ef	8.13 ± 0.84 ef	14.99 ± 1.51 d	29.07 ± 1.49 b
1097	(*E*)-4,8-dimethyl-l,3,7-nonatriene	0.22 ± 0.01 ef	0.14 ± 0.01 f	0.46 ± 0.07 cdef	0.76 ± 0.14 bc	1.31 ± 0.30 a	1.31 ± 0.28 a	0.91 ± 0.06 b	0.81 ± 0.06 bc	0.65 ± 0.05 bcd	0.54 ± 0.07 bcde	0.42 ± 0.0 6cdef	0.32 ± 0.06 def
1110	butyl tiglate	0.77 ± 0.21 a	0.38 ± 0.11 b	0.49 ± 0.01 b	0.50 ± 0.12 b	-	-	-	-	-	-	-	0.28 ± 0.01 b
1121	alloocimene	0.26 ± 0.01 a	0.25 ± 0.02 ab	0.17 ± 0.01 c	-	-	-	-	-	-	-	-	0.23 ± 0.02 b
1124	camphor	0.18 ± 0.02 d	0.17 ± 0.01 d	0.27 ± 0.03 cd	0.33 ± 0.03 c	0.47 ± 0.05 b	0.69 ± 0.04 a	0.74 ± 0.01 a	0.69 ± 0.05 a	0.68 ± 0.01 a	0.54 ± 0.06 b	0.46 ± 0.02 b	0.35 ± 0.04 c
1144	2-ethylhexyl acetate	1.83 ± 1.58	2.23 ± 2.03	2.21 ± 2.08	<0.01	<0.01	<0.01	<0.01	<0.01	<0.01	<0.01	<0.01	<0.01
1153	epoxylinalol	<0.01	0.10 ± 0.01 c	0.17 ± 0.02 b	0.24 ± 0.04 a	<0.01	-	-	-	-	-	-	-
1157	menthol	0.65 ± 0.01 ef	0.47 ± 0.05 f	0.90 ± 0.15 ef	1.27 ± 0.22 def	2.10 ± 0.35 cd	3.66 ± 0.55 ab	4.51 ± 0.62 a	4.34 ± 0.54 a	3.30 ± 0.29 b	2.73 ± 0.07 bc	2.06 ± 0.16 cd	1.47 ± 0.10 de
1167	methyl salicylate	0.91 ± 0.02	1.18 ± 0.08	1.83 ± 0.75	2.44 ± 0.92	3.22 ± 1.29	3.19 ± 1.26	2.33 ± 0.84	2.57 ± 0.98	2.24 ± 0.86	1.89 ± 0.73	2.40 ± 0.01	2.18 ± 0.13
1176	α-terpineol	0.99 ± 0.24 bc	1.53 ± 0.37 bc	2.84 ± 0.98 bc	5.79 ± 2.22 a	3.54 ± 0.96 ab	1.18 ± 0.29 bc	0.76 ± 0.18 c	1.25 ± 0.43 bc	2.30 ± 0.88 bc	3.11 ± 1.14 abc	2.97 ± 0.82 bc	2.21 ± 0.49 bc
1183	decanal	0.13 ± 0.02 d	0.14 ± 0.02 d	0.22 ± 0.02 c	0.35 ± 0.05 b	0.45 ± 0.01 a	<0.01	<0.01	-	-	-	-	<0.01
1196	benzenepropanol	0.13 ± 0.01 a	0.14 ± 0.01 a	0.14 ± 0.01 a	-	-	-	-	-	-	-	-	-
1209	2-ethylhexyl acrylate	0.52 ± 0.36	0.56 ± 0.39	0.84 ± 0.55	<0.01	<0.01	<0.01	<0.01	<0.01	<0.01	<0.01	<0.01	<0.01
1216	β-citral	0.91 ± 0.24	1.02 ± 0.28	1.58 ± 0.55	2.08 ± 0.78	1.10 ± 0.28	0.62 ± 0.02	<0.01	1.30 ± 0.01	1.74 ± 0.65	2.19 ± 0.75	2.09 ± 0.69	1.84 ± 0.59
1236	nerol	0.12 ± 0.01 a	0.11 ± 0.01 a	-	-	-	-	-	-	-	-	-	-
1244	geranial	0.29 ± 0.04 b	0.23 ± 0.06 c	0.25 ± 0.03 c	<0.01	-	-	-	-	-	0.53 ± 0.03 a	0.46 ± 0.05 a	0.47 ± 0.04 a
1300	tridecane	0.18 ± 0.03 e	0.23 ± 0.05 e	0.35 ± 0.07 de	0.55 ± 0.12 bcde	0.76 ± 0.17 abc	0.99 ± 0.24 a	0.83 ± 0.15 abc	0.90 ± 0.18 ab	0.81 ± 0.17 abc	0.66 ± 0.11 abcd	0.54 ± 0.12 bcde	0.46 ± 0.09 cde
1367	benzyl 3-methylbutanoate	0.22 ± 0.01 c	0.26 ± 0.08 bc	0.31 ± 0.03 ab	<0.01	-	-	-	-	-	-	0.28 ± 0.01 bc	0.39 ± 0.02 a
1380	copaene	0.33 ± 0.07 e	0.39 ± 0.11 de	0.60 ± 0.17 cde	0.87 ± 0.25 abcd	1.17 ± 0.38 ab	1.38 ± 0.02 a	1.11 ± 0.03 abc	1.26 ± 0.04 ab	0.81 ± 0.15 bcde	0.66 ± 0.05 cde	0.51 ± 0.05 de	0.41 ± 0.04 de
1415	isopentyl benzoate	0.12 ± 0.01 d	0.18 ± 0.01 c	0.26 ± 0.01 b	0.30 ± 0.01 a	-	-	-	-	-	-	-	0.19 ± 0.01 c
1431	β-caryophyllene	1.23 ± 0.36	1.54 ± 0.44	1.98 ± 0.56	2.67 ± 0.85	4.05 ± 1.41	4.47 ± 1.53	3.33 ± 0.90	4.69 ± 1.48	4.24 ± 1.19	3.50 ± 0.69	2.89 ± 0.56	2.28 ± 0.47
1446	(*Z*)-β-farnesene	0.94 ± 0.41	0.94 ± 0.38	1.30 ± 0.50	1.62 ± 0.65	1.86 ± 0.76	2.24 ± 0.05	1.66 ± 0.06	2.17 ± 0.09	1.79 ± 0.67	1.56 ± 0.51	1.35 ± 0.47	1.19 ± 0.41
1477	benzyl tiglate	0.97 ± 0.33	1.31 ± 0.36	1.82 ± 0.50	1.21 ± 0.22	0.53 ± 0.02	<0.01	0.54 ± 0.04	<0.01	<0.01	0.57 ± 0.02	0.68 ± 0.05	0.97 ± 0.19
1498	β-bisabolene	0.38 ± 0.01 d	0.38 ± 0.01 d	0.52 ± 0.02 c	0.74 ± 0.05 b	0.89 ± 0.05 a	<0.01	-	-	-	<0.01	<0.01	0.46 ± 0.01 cd
1547	nerolidol	5.69 ± 2.57	6.21 ± 2.57	7.61 ± 3.02	8.78 ± 3.68	9.57 ± 4.08	7.30 ± 3.01	4.85 ± 1.92	5.17 ± 2.09	5.41 ± 2.19	4.70 ± 1.74	4.96 ± 1.88	5.04 ± 1.90
1582	2-methylpropanoate	0.26 ± 0.01 c	0.20 ± 0.05 c	0.26 ± 0.03 c	0.50 ± 0.04 a	0.52 ± 0.03 a	-	-	-	-	-	-	0.34 ± 0.01 b

RI: Retention indices; ^Z^ Different letters within a column indicate significant differences at *p* < 0.05 by LSD multiple range. Data are mean ± SD of three replicates.

**Table 3:** Classification of the volatile components in different parts of *Onc.* Rosy Sunset.

Part	Component													
	Aldehydes		Alcohols		Esters		Hydrocarbons		Ketones		(Ep) Oxides		Total	
	Sum	RC ^y^	Sum	RC	Sum	RC	Sum	RC	Sum	RC	Sum	RC	Sum	RC
sepal	5	1964 a ^Z^	10	8675 a	11	1026 a	13	884 a	2	36 a	2	213 a	43	12798 a
petal	5	1981 a	10	7337 a	11	876 a	13	690 a	2	45 a	2	191 a	43	11120 a
lip	2	280 b	7	197 b	4	18 b	10	291 b	2	21 a	2	52 b	27	859 b
column	2	22 b	5	155 b	5	33 b	9	409 b	3	22 a	1	75 b	25	716 b

^Z^ Different letters within a column indicate significant differences at *p* < 0.05 by LSD multiple range. ^y^ RC: Relative content.
